# Monocyte and neutrophil levels are potentially linked to progression to IPF for patients with indeterminate UIP CT pattern

**DOI:** 10.1136/bmjresp-2021-000899

**Published:** 2021-11-18

**Authors:** Andrew Achaiah, Amila Rathnapala, Andrea Pereira, Harriet Bothwell, Kritica Dwivedi, Rosie Barker, Rachel Benamore, Rachel K Hoyles, Valentina Iotchkova, Ling-Pei Ho

**Affiliations:** 1 MRC Human Immunology Unit, University of Oxford, Headington, Oxford, UK; 2 Interstitial Lung Disease Service, Oxford University Hospitals NHS Foundation Trust, Oxford, UK; 3 Centre for Respiratory Medicine, Oxford University Hospitals NHS Foundation Trust, Oxford, UK; 4 Thoracic Radiology Department, Oxford University Hospitals NHS Foundation Trust, Oxford, UK; 5 Computational Biology and Statistics Unit, Weatherall Institute of Molecular Medicine, Radcliffe Department of Medicine, University of Oxford, Headington, Oxford, UK

**Keywords:** interstitial fibrosis, innate immunity

## Abstract

**Rationale:**

Idiopathic pulmonary fibrosis (IPF) is a progressive fibrotic lung disease with poor prognosis. Identifying patients early may allow intervention which could limit progression. The ‘indeterminate for usual interstitial pneumonia’ (iUIP) CT pattern, defined in the 2018 IPF guidelines, could be a precursor to IPF but there is limited data on how patients with iUIP progress over time.

**Objective:**

To evaluate the radiological progression of iUIP and explore factors linked to progression to IPF.

**Methods:**

We performed a retrospective analysis of a lung fibrosis clinic cohort (n=230) seen between 2013 and 2017. Cases with iUIP were identified; first ever CTs for each patient found and categorised as 'non-progressor' or 'progressors' (the latter defined as increase in extent of disease or to 'definite' or 'probable' UIP CT pattern) during their follow-up. Lung function trends, haematological data and patient demographics were examined to explore disease evolution and potential contribution to progression.

**Results:**

48 cases with iUIP CT pattern were identified. Of these, 32 had follow-up CT scans, of which 23 demonstrated progression. 17 patients in this cohort were diagnosed with IPF over a mean (SD) period of 3.9 (±1.9) years. Monocyte (HR: 23, 95% CI: 1.6 to 340, p=0.03) and neutrophil levels (HR: 1.8, 95% CI: 1.3 to 2.3, p<0.001), obtained around the time of initial CT, were associated with progression to IPF using Cox proportional hazard modelling.

**Conclusion:**

53% of our evaluable patients with iUIP progressed to IPF over a mean of 4 years. Monocyte and neutrophil levels at initial CT were significantly associated with progression in disease. These data provide a single-centre analysis of the evolution of patients with iUIP CT pattern, and first signal for potential factors associated with progression to IPF.

**Key message**

- How does the ‘indeterminate for usual interstitial pneumonia’ (iUIP) interstitial CT pattern evolve over time and what factors are associated with progression to definite and probable UIP pattern.
- In this retrospective single-centre analysis, 53% of evaluable cases with iUIP on initial CT scan progressed to probable or definite UIP CT pattern over an average of 4 years. Monocyte and neutrophil levels performed around the time of initial CT were significantly associated with progression to definite and probable UIP pattern.
- We discuss the implications of these findings, its strengths and limitations.

## Background

Idiopathic pulmonary fibrosis (IPF) is a progressive fibrotic condition characterised by a distinctive fibrotic pattern on thoracic CT scans, referred to as 'Usual Interstitial Pneumonia' (UIP). Despite advances in treatment, prognosis remains poor with a median survival of 2–4 years from diagnosis.^1^ Identifying and treating patients earlier could improve outcome.

It has been long acknowledged that there is a group of patients with subclinical interstitial lung disease (ILD). The term 'interstitial lung abnormalities' (ILA) was originally coined to define the spectrum of radiological patterns seen in these patients.^2^ ‘Indeterminate for usual interstitial pneumonia’ (iUIP) is one of these ILA subtypes. iUIP CT pattern is defined by presence of subtle reticulation, in the absence of honeycombing and traction bronchiectasis, with or without mild ground-glass opacification in a basal and subpleural distribution. The term was included in the 2018 ATS/ERS/JRS/ALAT IPF guideline to categorise CT features that do not meet the criteria for ‘definite’ or ‘probable’ UIP, and where there is no alternative ILD diagnosis.^3^ Prior to the 2018 guideline, cases compatible with the current iUIP and 'probable UIP' definition were collectively categorised as ‘possible UIP’.^4^ This was a highly heterogenous group, and many cases were subjected to surgical lung biopsies^5^ for clarification of diagnosis. Data accumulated from these biopsies suggest that in a significant number of patients with possible UIP pattern and particularly those with traction bronchiectasis, there is a reasonably good association with a histology diagnosis of UIP.^6 7^ As a result, the 2018 guidelines specify that where there is traction bronchiectasis, 'possible UIP' CT patterns should be placed into the category of 'probable UIP'. This leaves those with no traction bronchiectasis as 'iUIP' with features described above. Little is known of how radiographic iUIP progresses, although a study showed that up to 30% of cases that were biopsied demonstrated a histology pattern of UIP.^6^ This indicates that at least a proportion of patients with iUIP progresses to a clinical diagnosis of IPF. It is not clear which factors are associated with this.^8^

In this retrospective cohort study, we evaluated the radiological and clinical progression of patients with the iUIP CT pattern in one ILD centre, dividing cases into 'non-progressors' and 'progressors' (to 'probable' and 'definite' UIP pattern on CT by 2018 criteria or in extent of fibrosis on CT). We explored the association between blood neutrophil, monocytes and lymphocyte levels near to the point of first CT and patient demographics with progression. We found that 53% of evaluable cases progressed to a CT pattern of 'probable' or 'definite UIP' (all with IPF clinical diagnosis) within 4 years (mean/SD of 3.9/1.5 years) of initial CT. Using Cox proportional hazard analysis, we found that neutrophil and monocytes (but not lymphocytes), measured within 3 months of initial CT, significantly correlated with progression of iUIP in extent, and to a diagnosis of IPF.

## Methods

### Study design

We first, identified patients with iUIP CT patterns among our lung fibrosis cohort of patients who attended the Oxford Interstitial Lung Disease Service between 2013 and 2017. All patients in this lung fibrosis cohort had a CT pattern of 'possible UIP' or 'UIP' according to 2011. Radiologist reports for all available thoracic CTs for these patients (including first CTs before 2013) and up to August 2019 were reanalysed and cross-checked with reports from ILD multidisciplinary team (MDT) meetings, and recategorised according to the 2018 IPF guideline.^3^ Patients with iUIP CT patterns were then grouped as either ‘non-progressive’ or ‘progressive’ based on comparison of their first CT (including those prior to attendance at Oxford) to the latest follow-on CT (up to cut-off point of August 2019). We defined ‘non-progressive’ as no change in CT scan in terms of extent of disease or change in pattern of disease; and ‘progressive’ if there were either visual (qualitative) increase in extent of disease or progression of CT pattern to 'definite' or 'probable UIP' pattern.

The following data were collected—patient demographics, dates of first CT and follow-on CTs, year of diagnosis of IPF (by ILD MDT meetings, and according to 2018 guidelines), all available pulmonary function tests, comorbidities, neutrophils, lymphocytes and monocyte levels performed using standard hospital 'full blood count' analysis and clinical outcomes including disease progression, survival and years of clinic follow-up. Indications for follow-on CT were also recorded.

### Patient and public involvement

Patient and public were not involved in design, recruitment or conduct of this study.

### CT scan and analysis

CT scans were acquired using a 64-detector row CT scanner (LightSpeed VCT XT; GE Medical Systems, Milwaukee, Wisconsin, USA). Images were reconstructed using a high spatial resolution algorithm. All CT abnormalities were defined and analysed using standard Fleischner-based terminology.^9^

### Statistical analysis

Where data are expressed as means, SD are shown. Tests for normality of data were performed using a D'Agostino and Pearson test and following this the difference between groups was analysed using Student’s t-tests or Mann-Whitney test for parametric and non-parametric analysis, respectively. Contingency tests (Fisher’s exact test of significance) were used to assess categorical data. Survival analysis (log-rank test of significance) was performed to evaluate time-to-mortality. For analyses of correlates for progression to IPF (2018 criteria),^2^ the Cox proportional hazard modelling to determine HRs was employed, testing their significance in two settings: (A) using data from patients regardless of whether they proceeded to defined events ('definite' or 'probable' UIP pattern on CT or progression in extent of disease) but up to August 2019 (n=32) and (B) restricting the patients to only those that did progress to 'definite' or 'probable' UIP pattern on CT during the period of analysis (n=17). HRs generated for continuous covariates represent the change in the risk of outcome if the covariate in question changes by one unit. HRs generated for dichotomised covariates represents the risk of achieving outcome if the covariate is present. All analyses were performed using Graphpad Prism (V.8) with the exception of Cox proportional hazard modelling which was performed with R Studio (V.3.6.2) statistical programming language packages Survival (V.3.1.8) and Survminer (V.0.4.6) by our statistical team (led by VI). We used the cox.zph function in the R package survival for testing the proportional hazards assumption of a Cox regression. Statistical significance was performed using the likelihood ratio test (preferred compared with Wald test due to our smaller number) as reported by the coxph function at the 95% significance level. Reported p values were two-sided and a p value<0.05 was considered significant.

## Results

### Progress of iUIP patients from point of initial CTs

Of the 230 individual patients who attended the lung fibrosis ILD clinic between 2013 and 2017 with CT patterns of 'probable', 'possible' or 'definite UIP’, 48 (21%) cases with iUIP pattern were found. Thirty-two of these patients had at least one follow-on thoracic CT from the first scan. In the 16 cases that did not have a follow-on scan (and therefore could not be categorised into progressors or non-progressors), 13 cases were discharged after a mean of 2.1 years without a CT, as they were clinically stable and three were deemed not to require a second CT scan clinically.

**Figure 1:**
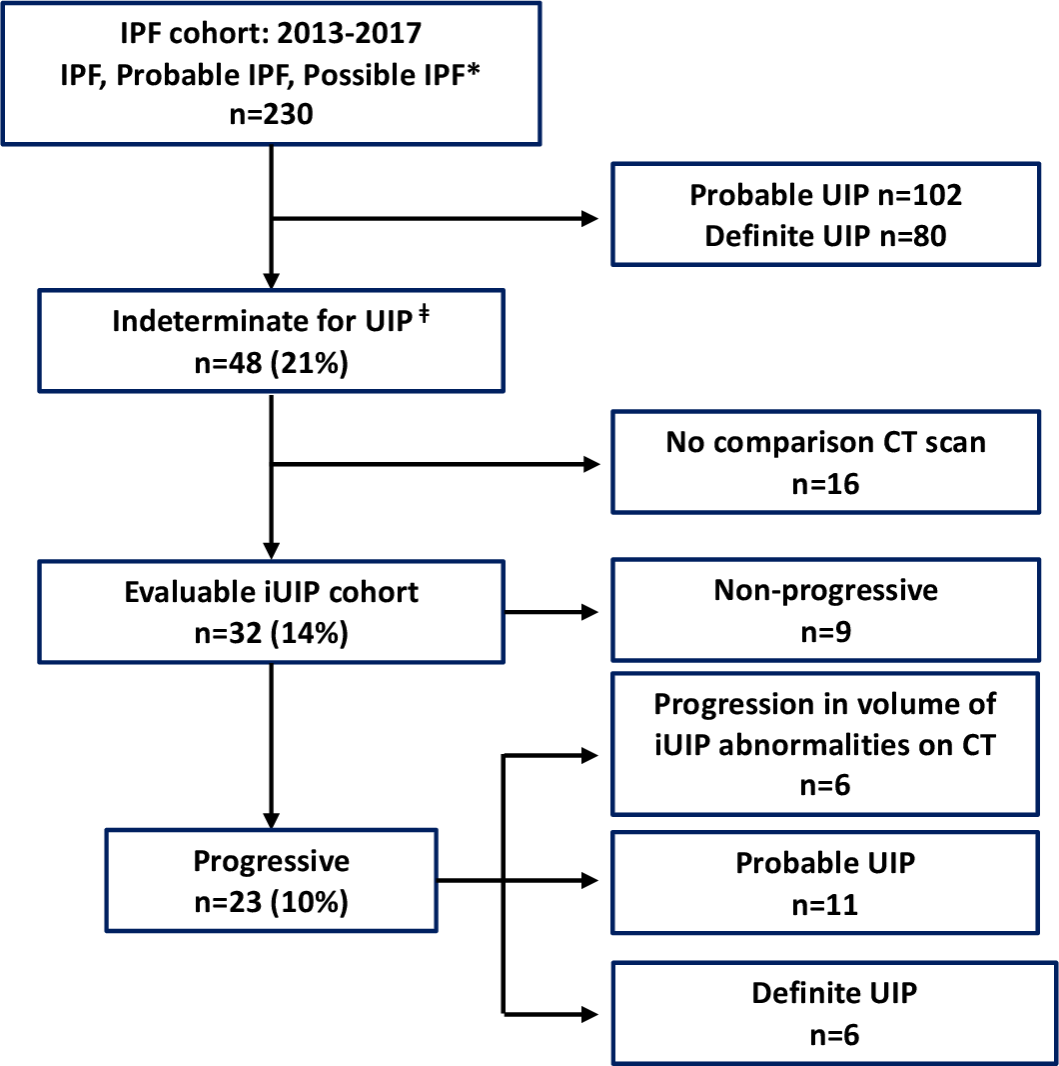
Flow diagram of radiographic progression of UIP within the IPF cohort (n=230). Clinical diagnosis *as per 2011 IPF guideline^4^; ǂas per 2018 IPF guideline.^3^ IPF, Idiopathic pulmonary fibrosis; iUIP, indeterminate for UIP; UIP, usual interstitial pneumonia.

Of the 32 patients who had follow-on scans, 9 (28%) patients showed no change in CT pattern or extent in disease over a mean (±SD) of 2.1±0.9 years and were classified as non-progressors. The most frequent indications for follow-on CT in this group was for nodule surveillance (64%) and to identify any further radiographic progression of iUIP (14%). Twenty-three (72%) demonstrated progression. Six showed increase in extent of iUIP but no change in pattern over 3.1±0.8 years; 11 progressed to 'probable UIP' over 3.8±1.6 years and 6 to 'definite' UIP over 4.1±2.4 years. For the 23 ‘progressors’ the most frequent indication for follow-on CT was to investigate worsening symptomatic breathlessness (52%) and decline in lung function parameters (28%). All those who progressed to 'definite' and 'probable UIP' were diagnosed clinically, with IPF after discussion in the ILD MDT meetings; five of which underwent surgical lung biopsy to attain definitive diagnosis. Therefore 53% (17 of 32) of our evaluable iUIP cohort (ie, those who had follow-on CTs) or 35% (17 of 48) of all patients with iUIP (if those who did not have a follow-on CT were included) progressed to a clinical diagnosis of IPF over a mean period of 3.9±1.9 years. These findings are summarised in figure 1.

**Figure 2:**
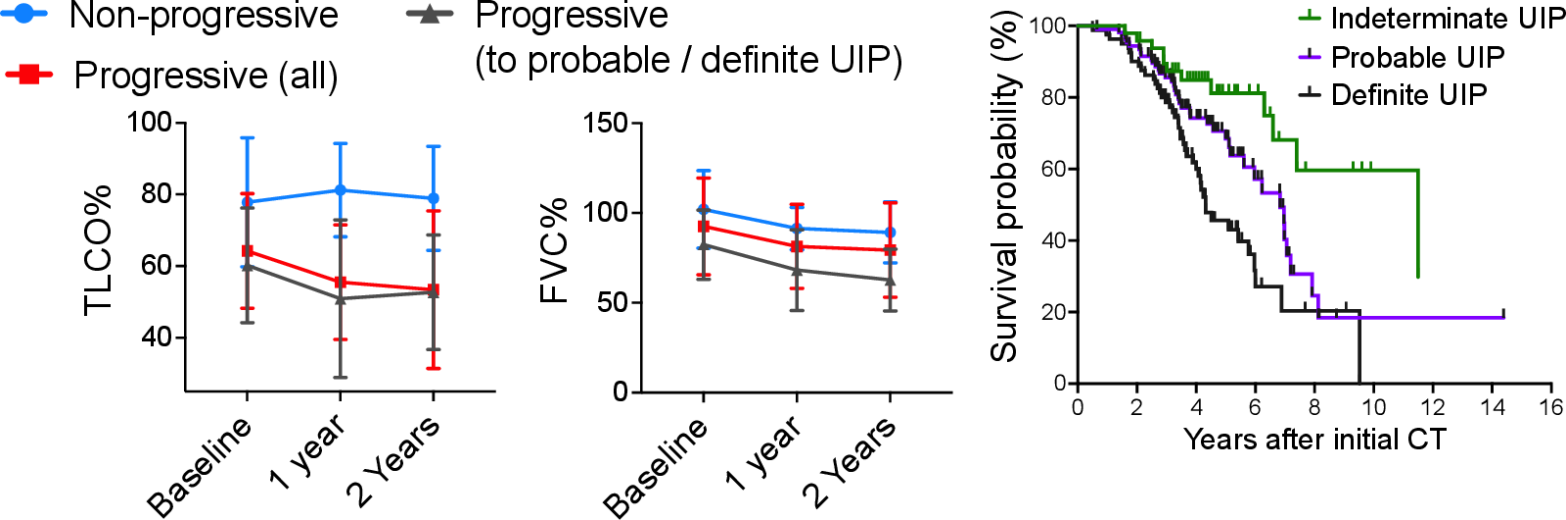
Lung function progression from baseline (within 3 months of initial CT scan) for non-progressors, those who progressed in amount of disease and to 'definite' and 'probable' UIP ('progressors (all)'), and those who progressed to definite and probable UIP only ('progressors (to probable/definite UIP)'). Mean (SD) values are displayed; no statistical analyses were performed. Survival curve for all patients divided into those with iUIP, definite and probable UIP on thoracic CT scan at their first CT scan in the study. FVC, forced vital capacity; iUIP, indeterminate for UIP; TL_CO_, carbon monoxide transfer factor; UIP, usual interstitial pneumonia.

Twelve (25%) of 48 iUIP cases died during follow-up. The mean time from initial CT reporting iUIP to all-cause mortality was 4.6±2.9 years. Respiratory-related deaths were confined to the progressive iUIP group; these accounted for six of the nine deaths in this group: two to pneumonia and four to end-stage IPF. There was a trend to a greater number of hospitalisation events (39% vs 22% (OR: 2.25, 95% CI: 0.40 to 12.32)) and greater smoking history (86% vs 67% (OR: 3.2, 95% CI: 0.59 to 15.9)) in the progressive iUIP group (table 1). Forced vital capacity (FVC) and carbon monoxide transfer factor (TL_CO_) values at initial CT were not different between the progressor and non-progressor groups. However, at 1 year from initial CT, mean change in FVC for the 'non-progressor' group was −0.03 (±0.26) litres versus −0.26 (±0.39) litres in the 'progressive' group; p=0.16 (figure 2A). Median survival for patients with an initial CT of iUIP was also significantly better than patients with a first CT demonstrating either 'probable' or 'definite UIP' (figure 2B). Demographic data, physiological indices and comorbidity profiles in the progressor and non-progressor iUIP groups are shown in table 1.

**Table 1:** Characteristics for patients with iUIP who had at least two CT scans (n=32), at the point of initial CT when iUIP was identified

	Non-progressive iUIP	Progressive iUIP	P value or OR	95% CI
n	9	23		
Male	6 (66%)	18 (78%)	1.8	0.37 to 8.34
Female	3 (33%)	5 (22%)	0.6	0.12 to 2.64
Age at first CT showing iUIP (±SD)	76.7 (±6.2)	72.3 (±8.6)	p=0.277	–
Never smoker	3 (33%)	3 (14%)	0.3	0.06 to 1.70
Ex-smoker	6 (67%)	19 (86%)	3.2	0.59 to 15.9
Respiratory comorbidity	1 (11%)	8 (40%)	5.3	0.61 to 65.6
Cardiac comorbidity	6 (66%)	17 (85%)	1.3	0.27 to 7.52
Diabetes mellitus	4 (44%)	3 (15%)	0.2	0.05 to 1.17
TL_CO_ (mmol/min/kPa)	5.0 (±0.9)	5.3 (±1.7)	p=0.983	–
%TL_CO_	77.8 (±18.1)	64.2 (±16.0)	p=0.077	–
FVC (l)	2.90 (±0.7)	3.24 (±1.1)	p=0.728	–
%FVC	102.00 (±21.6)	92.6 (±26.9)	p=0.285	–
FEV_1_ (l)	2.33 (±0.7)	2.36 (±0.7)	p=0.853	–
%FEV_1_	98.4 (±18.8)	85.9 (±19.4)	p=0.362	–
CPI score	67.7 (±18.3)	69.5 (±10.4)	p=0.327	–

Data are divided into progressor and non-progressor groups. % in parenthesis is proportion of specified group.

Statistical analysis expressed at p value or OR with 95% CI. Lung function parameters refer to those measured within 3 months of first CT scan. CPI as calculated by Wells *et al*.^18^

CPI, Composite Physiological Index; FEV_1_, forced expiratory volume in one second; FVC, forced vital capacity; iUIP, indeterminate for UIP; TL_CO_, carbon monoxide transfer factor; UIP, usual interstitial pneumonia.

### Monocyte and neutrophils but not lymphocyte levels within 3 months of initial CT scan are associated with progression of iUIP to definite or probable UIP CT patterns

Having characterised these subgroups, we explored potential predictor variables for progression to IPF.

A univariate analysis of the following variables (taken at the point of diagnosis (initial CT scan)) age, gender, FVC, smoking status, monocyte count (continuous, median value or dichotomised at > and <0.9×10^9^/L), lymphocyte count (continuous, median value < and >1.0×10^9^/L) and neutrophil count (continuous, median value, < and >7.5×10^9^/L) were undertaken using the Cox proportional hazard modelling method. Dichotomised values were selected from the upper limit of normal range for neutrophils and lymphocytes, and from Scott *et al* paper for monocytes.^10^

We determined the HRs for progression and tested their significance (using likelihood ratio test) in two settings using data from (A) all evaluable cases (n=32) and (B) only patients who progressed to IPF during the period of analysis (n=17). Apart from smoking status none of the variables violated the proportional hazards assumption of a Cox regression in setting A. In setting B lymphocyte count (continuous) did, while smoking status did violate the proportional hazards assumption of a Cox regression (table 2). Output from smoking status in setting A and lymphocytes in setting B were therefore not used.

**Table 2:** Univariate COX proportional hazard analysis of cohort

	Beta	HR (95% CI for HR)	Likelihood ratio test	Likelihood ratio test P value	P value PH assumption (<0.05 indicates violation)
Univariate Cox PH analysis on all patients (n=32): setting A					
Gender male vs female	1.20	3.3 (0.71 to 15)	3.00	0.08	0.52
Monocytes (×10^9^/L)	3.10	23 (1.6 to 340)	4.80	0.03*	0.26
Monocytes (>0.9×10^9^/L)	1.40	3.9 (1.3 to 12)	4.90	0.03*	0.40
Monocytes (median)	1.30	3.8 (1.2 to 13)	5.70	0.02*	0.70
Lymphocytes (×10^9^/L)	0.47	1.6 (0.64 to 4)	0.98	0.32	0.05
Lymphocytes (<1.0×10^9^/L)	0.80	2.2 (0.28 to 18)	0.47	0.49	0.24
Lymphocyte (median)	0.39	1.5 (0.51 to 4.2)	0.52	0.47	*0.002*
Neutrophil (×10^9^/L)	0.57	1.8 (1.3 to 2.3)	18.00	2.0×10^−5*^	0.29
Neutrophil (>7.5×10^9^/L)	3.80	43 (4.2 to 430)	12.00	0.00065*	0.06
Neutrophil (median)	1.40	4.1 (1.3 to 12)	6.60	0.01*	0.85
FVC (%predicted) at initial CT	−0.02	0.98 (0.95 to 1)	3.40	0.07	0.60
Smoking (never vs ex)	−0.43	0.65 (0.17 to 2.5)	0.37	0.54	*0.03*
Age at initial CT	−0.02	0.98 (0.92 to 1)	0.32	0.57	0.57
Univariate Cox PH models on patients who progressed to IPF in analysis period (n=17): setting B					
Gender male vs female	0.85	2.3 (0.52 to 10)	1.50	0.22	0.84
Monocytes (×10^9^/L)	3.50	33 (1.8 to 600)	5.50	0.02*	0.73
Monocytes (>0.9×10^9^/L)	1.10	3 (0.95 to 9.7)	3.20	0.07	0.67
Monocytes (median)	1.40	3.9 (1.2 to 13)	5.60	0.02*	0.88
Lymphocytes (×10^9^/L)	0.32	1.4 (0.49 to 3.8)	0.37	0.55	*0.002*
Lymphocytes (<1.0×10^9^/L)	2.70	15 (0.97 to 250)	3.00	0.09	0.16
Lymphocyte (median)	−0.05	0.95 (0.33 to 2.7)	0.01	0.93	*0.003*
Neutrophil (×10^9^/L)	0.43	1.5 (1.2 to 2)	9.70	0.002*	0.44
Neutrophil (>7.5×10^9^/L)	3.00	20 (2 to 200)	7.80	0.005*	0.06
Neutrophil (median)	1.10	3.1 (1 to 9.7)	4.30	0.04*	0.89
FVC (% predicted) at initial CT	−0.003	1 (0.97 to 1)	0.06	0.81	0.61
Smoking (never vs ex)	−0.42	0.65 (0.17 to 2.5)	0.36	0.55	0.08
Age at initial CT	0.03	1 (0.96 to 1.1)	0.66	0.42	0.60

FVC, forced vital capacity; IPF, idiopathic pulmonary fibrosis.

**Figure 3:**
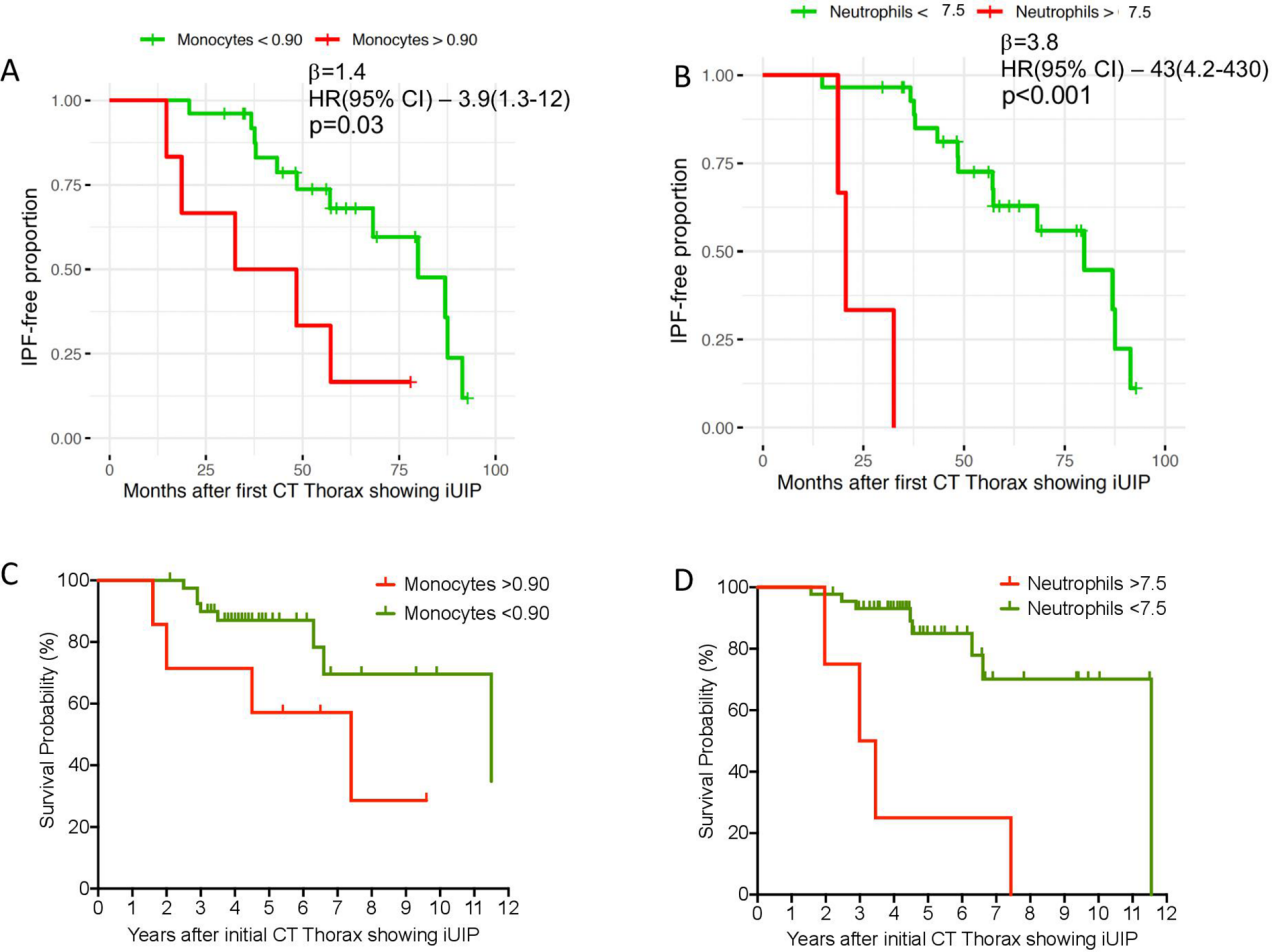
iUIP-free months in all patients (n=32) with (A) Monocytes levels>and < 0.9x10^9^/L and (B) neutrophils > and <7.5×10^9^/L at the point of initial CT with iUIP (univariate COX proportional hazard analysis of all patients with iUIP on initial CT scan; P value analysed by likelihood ratio test). (C) Survival curve for all patients with iUIP at initial scan (n=48) divided with (A) monocytes > and <0.9×10^9^/L and (D) neutrophils > and <7.5×10^9^/L, regardless of progression, and including those without second CT scan. iUIP, indeterminate for UIP; UIP, usual interstitial pneumonia.

In both settings A and B, we found that increased neutrophils and monocytes (both binary and continuous variables) were associated with progression within the follow-up period— monocytes (continuous) β=3.10; HR (95% CI)=23 (1.6–340), p=0.03; neutrophils (continuous) β=0.57; HR (95% CI)=1.8 (1.3–2.3), p<0.001 (table 2; and figure 3A, B). Apart from blood leukocytes, no other variables showed significant difference. Histograms for the distribution of monocyte, neutrophil and lymphocyte values are shown in supplementary data (online supplemental figure S1).

10.1136/bmjresp-2021-000899.supp1 Supplementary data

Due to the low numbers and some high monocyte, lymphocyte and neutrophil counts, we also checked the sensitivity of HR and their significance using a more balanced design by splitting the full blood cell counts by their median values. The statistical significance and direction of effect in both settings A and B were maintained (table 2).

We also modelled the binary monocyte, lymphocytes and neutrophil levels to account for the covariates of gender, age and FVC in the Cox PH multivariate mode. This multivariate analysis also preserved the significance of monocyte and neutrophil count in settings A and B (table 3). Significance of individual covariates was reported using Wald test and overall significance of the model (compared with the alternative of no effect of any covariate) was tested using likelihood ratio test as reported by the coxph function in R.

**Table 3:** Multivariate COX proportional hazard analysis of cohort

	Beta	HR (95% CI for HR)	Wald test p value	P value PH assumption (<0.05 indicates violation)
Multivariate Cox PH analysis on all patients (n=32) (setting A)				
Gender male vs female	0.64	1.90 (0.35 to 10)	0.46	0.92
Age at initial CT	−0.07	0.93 (0.85 to 1)	0.15	0.84
Smoking (never vs ex)	−2.30	0.10 (0.015 to 0.72)	0.02*	0.09
FVC (% predicted) at initial CT	−0.04	0.96 (0.92 to 1)	0.05*	0.71
Monocytes (>0.9×10^9^ /L)	3.30	27 (2 to 370)	0.01*	0.09
Lymphocytes (<1.0×10^9^ /L)	−1.00	0.37 (0.013 to 10)	0.56	0.85
Neutrophil (>7.5×10^9^ /L)	3.50	35 (1.7 to 680)	0.02*	0.06
GLOBAL significance=likelihood ratio test 21 (p=0.004), PH assumption p=0.18				
Multivariate Cox PH models on patients who progressed to IPF in analysis period (n=17) (setting B)				
Gender male vs female	1.30	3.50 (0.52 to 24)	0.20	0.43
Age at initial CT	−0.14	0.87 (0.78 to 0.97)	0.02*	0.20
Smoking (never vs ex)	−3.40	0.03 (0.0028 to 0.42)	0.008*	0.37
FVC (% predicted) at initial CT	−0.05	0.95 (0.89 to 1)	0.07	0.23
Monocytes (>0.9×10^9^/L)	8.80	6700 (19 to 2400000)	0.003*	0.87
Lymphocytes (<1.0×10^9^/L)	−5.00	0.01 (4.7^−5^ to 1)	0.05	0.34
Neutrophil (>7.5×10^9^/L)	5.20	180 (2.3 to 14000)	0.02*	0.24
GLOBAL significance=likelihood ratio test 21 (p=0.004), PH assumption p=0.69.				

GLOBAL significance=likelihood ratio test 21 (p=0.004), PH assumption p=0.69.

FVC, forced vital capacity.

The adjusted HR and CIs were large but for all significant values, the lower limit range of the CI was above 1, providing confidence that the HR were above 1 for monocytes and neutrophils. We do not think the absolute values of the adjusted HR and upper CIs are an accurate reflection of patient risk, and are likely inflated due to the small number of patients.

Lymphocyte levels (binary or continuous) were not significantly associated with time to IPF diagnosis in any of the models.

Finally, we examined survival after categorising all cases, regardless of whether they had a follow-on CT (n=48), according to baseline monocyte count of > or <0.90×10^9^/L and neutrophils of > or <7.5×10^9^/L. We observed a trend towards shorter survival time for higher monocyte levels (HR: 2.9, 95% CI: 0.59 to 14.51, p=0.06 log-rank test, censoring event as death or August 2019 for survival group); and higher neutrophils (HR: 6.95, 95% CI: 0.72 to 66.7, p=0.0002 log-rank test) (figure 3C, D).

## Discussion

In this single-centre retrospective analysis of iUIP progression, we observed that among an unselected group of 230 patients followed up for lung fibrosis, the prevalence of those with iUIP CT pattern was 21%. A minimum of 35% (if we included those who did not have a second thoracic CT scan due to lack of need), progressed to IPF within 4 years, with 25% death in the follow-up period. These data suggest that iUIP CT pattern is an important entity, a precursor to IPF in some patients within a few years, and for these patients, there could only be a short period to intervene to prevent progression. Further analyses suggest that increased neutrophils and monocytes levels might identify this group of patients with higher risk of progression to IPF.

The prevalence of iUIP among patients seen in our IPF clinic is similar to the analysis from only one other study on prevalence. Diridollou *et al* recategorised 89 cases with 'possible UIP' CT pattern and found 17% of these were iUIP.^11^ In a large birth cohort study (AGES-Reykjavik, N=5320), Putman *et al* observed a 2.52% prevalence of patients with iUIP, and demonstrated that patients with iUIP CT pattern had a greater risk of mortality compared with those without any interstitial lung abnormality (p<0.0001 (HR: 1.6, 95% CI: 1.3 to 2.0)).^12^ Neither study examined how patients with iUIP progress in the ensuing years from initial diagnosis.

It is noteworthy from our analysis that there is no statistical difference between the starting FVC and TL_CO_ for the progressor and non-progressor groups of patients with iUIP; though there is a trend towards lower % predicted TL_CO_ (table 1). The calculated CPI score which adjusts for presence of emphysema is, not unexpectedly, also similar between the two groups. However, with univariate (but not multivariate) analysis (table 3), lower FVC at initial CT did (just) correlate with greater likelihood to progression for the individual patient, which is clinically cogent. These data suggest that factors other than severity of disease may be driving the progression of iUIP to IPF.

We chose to examine monocyte, neutrophils and lymphocyte levels, in part, because of the potential utility in clinic due to these being routine performed blood tests but primarily because of the possible link between monocytes and mechanism of disease, as shown in Scott *et al*^10^ and our own work.^13^ We used monocyte counts of >0.9×10^9^/L as Scott *et al* had identified this as the level of monocyte, above which was associated with higher mortality risk in patients with IPF. In Fraser *et al,*^13^ we observed that monocytes in patients with IPF showed type 1 interferon primed phenotype which could account for more robust and potentially injurious response to the alveolar epithelium when triggered, during for example, a viral infection. This study provides impetus to investigate the possibility that neutrophils (which were not investigated in Scott or Fraser studies) could also be involved.

There are several limitations to our study. The monocyte, neutrophil and lymphocyte levels were measured at one point (nearest to the CT scan). This is in keeping with work from other much larger studies^10^ but there is a risk that the values are not representative of the steady-state values, particularly in a necessarily small cohort as ours. In further studies, it will be useful to have repeated samples over 6–12 months to determine if the neutrophil, lymphocyte and monocyte values are representative for the patient, and reduce bias towards the possibility of levels linked to an infective episode, for example.

The most obvious limitation is the small number of patients. This probably reflects the relatively small proportion of iUIP scans that are referred for clinical assessment without delay. These patients are often asymptomatic in the early stages of their ILD.^14^ However, it is becoming increasingly recognised that a proportion of ILAs will progress to clinically significant ILD.^14^ Furthermore, identification of ILAs is predicted to increase with implementation of lung cancer screening and increased use of CT for other diagnostic purposes.^15^ This may increase reporting of iUIP in the future, making the need for biomarkers that can risk-stratify for progression even more pertinent.

In our retrospective study, not all follow-on CT scans included in analysis were undertaken in a uniform timeframe across the cohort. With exception of the lung nodule surveillance imaging most serial CTs were performed according to clinical indication and where there was concern for objective deterioration. A greater proportion of repeat CT scans were performed to investigate symptomatic change in the ‘progressor’ group, and this may have introduced bias towards detection of progression. We also acknowledge that length of follow-up is shorter in the non-progressor group, although not significant (p=0.06). However, it has been well documented that initial progression particularly during first 6–12 months is predictive of progression of fibrotic ILDs.^16 17^ Therefore a follow-up for 2 years or more, as seen in the majority of our cohort, may be sufficient to identify patients with a progressive phenotype.

The small sample size limits the power of this study and has contributed to some of the large HRs and CIs observed. Therefore, our findings are primarily indicative rather than definitive signals. The values of the HR cannot be interpreted as an absolute numerical risk but rather an indication that the risk exist and that it is statistically significant since the lower limit of the 95% CI is above 1. Consistent findings in proportional hazards modelling by both univariate and multivariate analyses also lend support to the contribution of monocytes and neutrophil levels to progression to IPF. As the numbers of patients with iUIP CT pattern disease are small, a multisite cohort will be required to confirm these findings. Nevertheless, our findings suggest that at least some patients with iUIP CT pattern could be patients with early IPF and their disease progression could be linked to higher levels of monocytes and neutrophils. Further studies could validate the use of blood monocytes and neutrophils as biomarkers for patients with iUIP and were at higher risks of progression to IPF and all-cause mortality.

## Data Availability

All data relevant to the study are included in the article or uploaded as supplementary information.
