# Supplementary figures and images for: Monocyte and neutrophil levels are potentially linked to progression to IPF for patients with indeterminate UIP CT pattern
Source: BMJ Open Respir Res. 2021 Nov 18;8(1):e000899. doi: 10.1136/bmjresp-2021-000899 (PMC8606778; doi:10.1136/bmjresp-2021-000899)

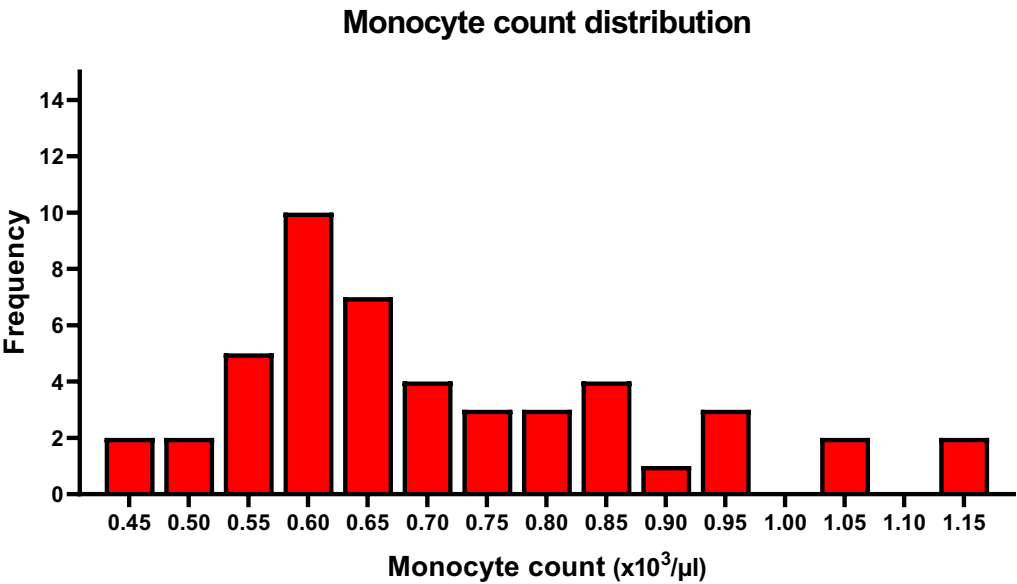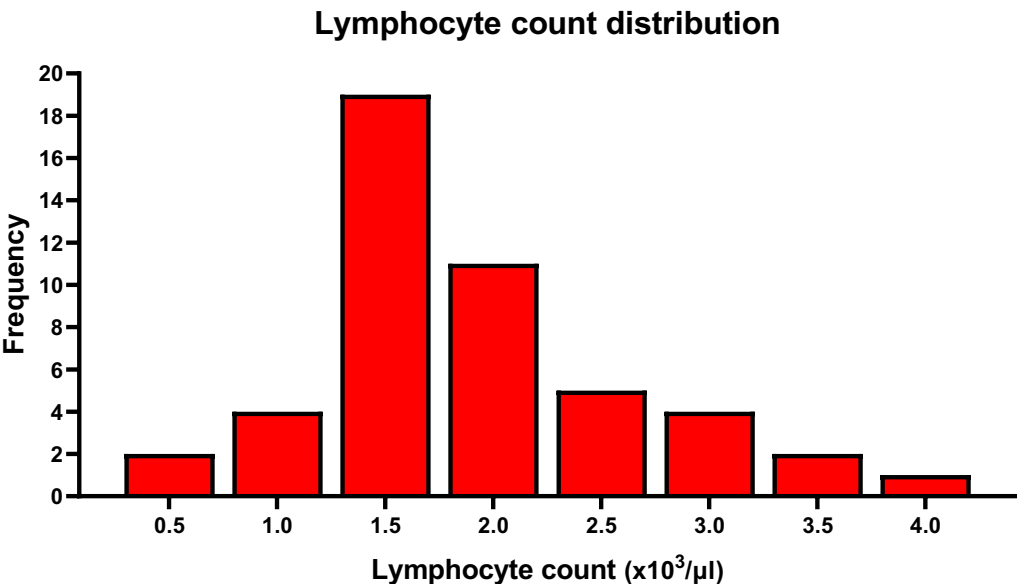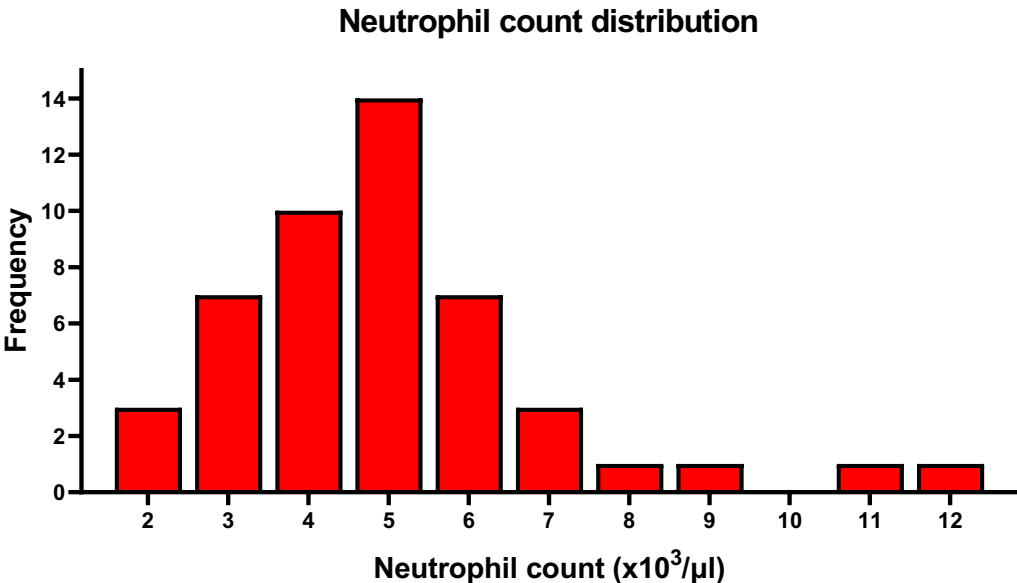

Supplementary Figure S1.  
Blood leukocyte distributions

Supplement: Supplementary data [file bmjresp-2021-000899supp001.pdf]
